# Giant Borderline Mucinous Cystadenoma: A Distressing Scenario

**DOI:** 10.7759/cureus.23968

**Published:** 2022-04-08

**Authors:** Asawari Deo, Deepti Shrivastava, Amardeep Shanoo

**Affiliations:** 1 Department of Obstetrics and Gynaecology, Jawaharlal Nehru Medical College, Datta Meghe Institute of Medical Science (Deemed to be University), Wardha, IND

**Keywords:** laparotomy, distention, ascites, mucinous tumors, giant ovarian tumors

## Abstract

Abdominal cystic tumors are highly prevalent. Due to the availability of superior imaging techniques, they are now diagnosed more commonly and considerably earlier. Although giant masses are seen sporadically, particularly in the rural scenarios. A 74-year-old multipara with distension of abdomen and breathlessness misdiagnosed as ascites initially was later on confirmed as giant borderline mucinous cystadenoma. We wish to highlight this case to bring forth the plight of rural women who are deprived of standard health care. The case of ovarian tumor presented seems to be the largest documented ovarian tumor in central India to the best of our knowledge.

## Introduction

The risk of getting diagnosed with an ovarian tumor is about 1% to 1.5% [1], although the diagnosis of ovarian cysts is fairly common, due to increased accessibility and routine use of newer imaging modalities. These cysts rarely get to gigantic proportions owing to the advances in radiological techniques, even in this era of modern medicine, rural women with lack of access to state of art health care, are still reporting cases which perhaps have led to sporadic mentions of it in literature [2], which more significantly reflects on the lack of health care and the plight of these women.

Cystic lesions with loculi lining that has mucin secreting epithelium, which appears like the endocervical glands and the gastrointestinal epithelium constitutes mucinous ovarian tumors [3]. Such tumors may have the potential to reach massive dimensions and can fill up the entire abdominal cavity. Benign mucinous cystadenomas make up roughly 15% of all ovarian neoplasms which is among the largest recorded tumors. About 80% of mucinous tumors remain benign, 10% constitute borderline and 10% are malignant, of which 80% are metastatic, with the gastrointestinal tract being the primary site in 45% of cases other organs such as the pancreas and breast have also been implicated. They are commonly found between the third and fifth decade of life [4].

Management of ovarian tumors essentially starts with an assessment of the malignant potential of the tumor by the risk malignancy index which depends on the menopausal status, ultrasonographic findings and levels of ca 125 [5]. Surgical interventions are based on the patient’s age, menopausal status, presence of any other medical or surgical illness, with open or laparoscopic approach. We present a case of septuagenarian lady who initially presented as case of massive ascites, which turned out to be giant ovarian mass which on histopathological examination was diagnosed as borderline mucinous cystadenoma.

## Case presentation

A 74-year-old woman, grand multipara, presented to the casualty of a tertiary Centre of a rural hospital in central India, in emergency hours with massive ascites and breathlessness (NYHA III), with a history of gradual abdominal distension over the past seven years, with sparse episodes of dull-aching pain in the abdomen, weight loss, and chronic constipation. On examination, the patient was cachexic and pale, with a weight of 75 kilograms, her pulse was 124 beats per minute, and blood pressure was recorded as 160/100 mmHg, with SpO_2_ 92% on room air.

**Figure 1:**
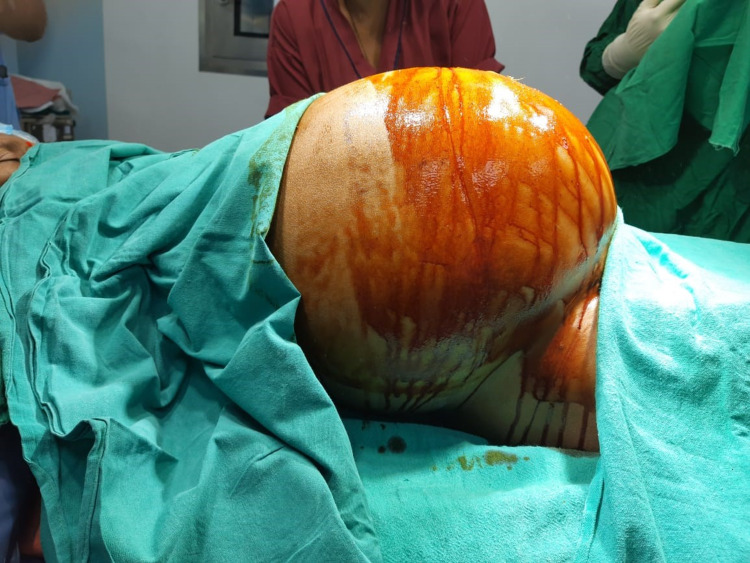
Preoperative distension of the abdomen and the extent of the tumor

On abdominal examination, the skin was tense and shiny with a uniformly large cystic mass extending from the pubis to the xiphisternum (Figure 1). There was uniform fullness in both the flanks, no tenderness was felt nor was any guarding or rigidity. On per speculum examination, the cervix appeared taken up and senile atrophic changes in the vagina were seen. On bimanual examination the size of the uterus could not be ascertained, fullness was felt in bilateral fornices. on laboratory investigation, her hemoglobin was 6.9g% and hematocrit was 28.4%, CA 125 was 53 U/mL and CA 19.9 was 1,000 U/mL, the rest of the other tumor markers CEA, B-HCG, AFP, and other laboratory investigations were within normal limits.

**Figure 2:**
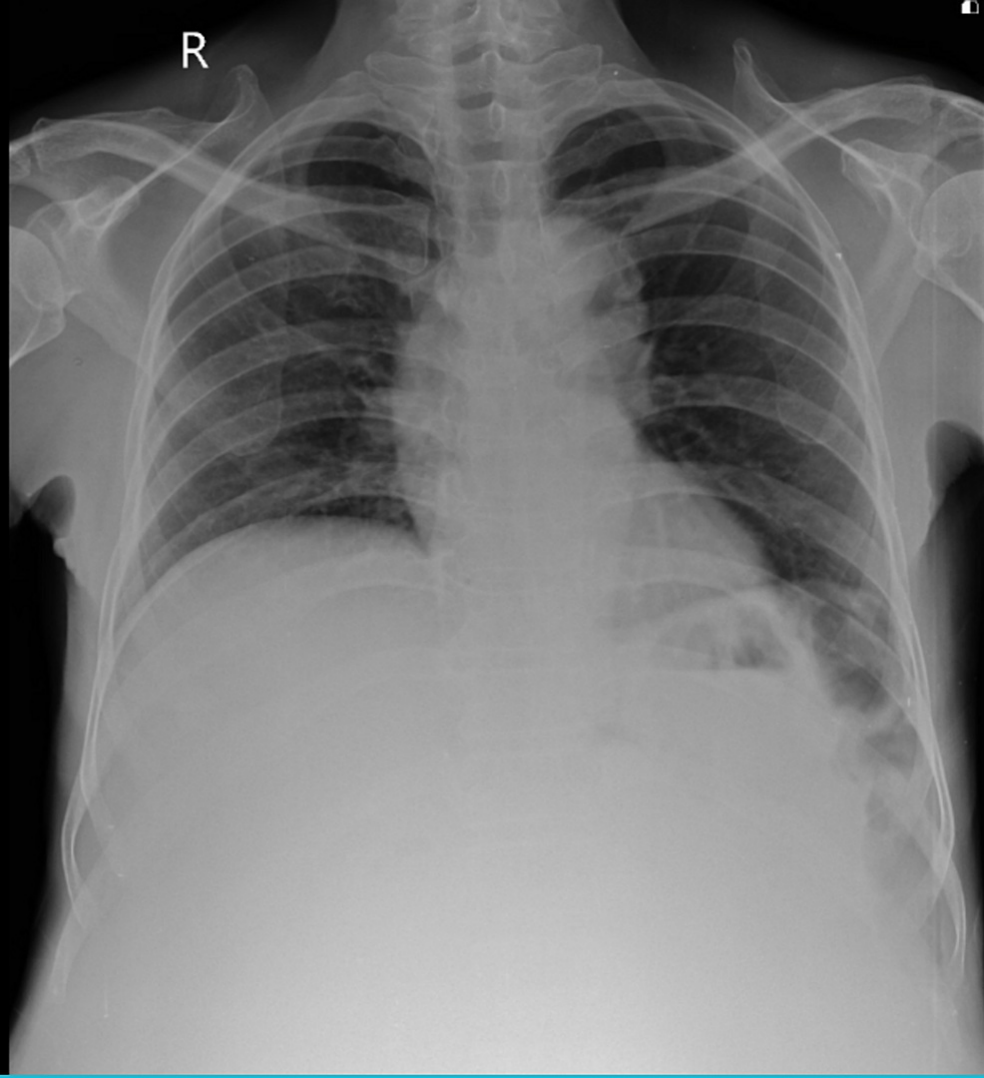
X-ray showing pushed-up diaphragm and obliterated costophrenic angle

Chest x-ray revealed obliteration of costophrenic angles and pushed up diaphragm (Figure 2). 2D-echo showed mild aortic regurgitation with left ventricular ejection fraction of 55%.

**Figure 3:**
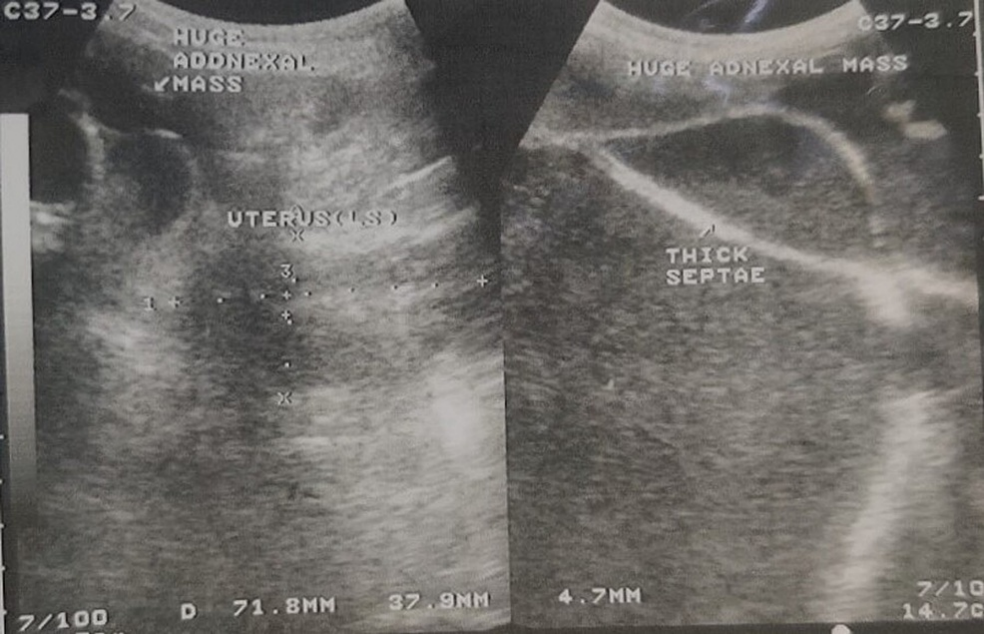
Greyscale transabdominal ultrasonographic picture showing multiloculated, solid cystic mass with thick septae (left and right)

**Figure 4:**
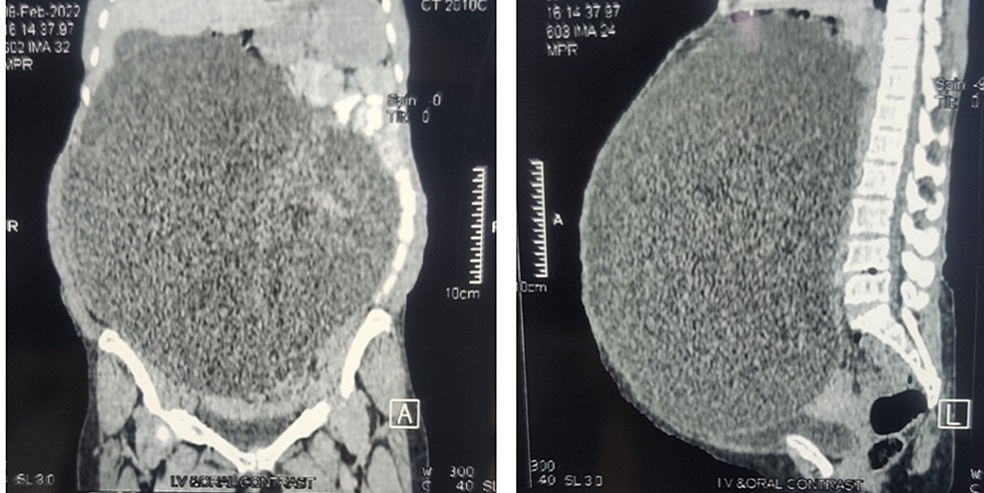
Multidetector CT of abdomen, sagittal (left) and axial (right) view showing mutiloculated cystic lesion arising from right ovary with thick septation *CT- Computed Tomography

Ultrasonography of abdomen and pelvis reported huge cystic mass having thickened wall (5.0 mm) with multiple septations and low-level echogenic foci, centrally positioned present in all nine quadrants of abdomen compressing the bowel arising from behind uterus and cephalad of urinary bladder, but anterior to the rectum (Figure 3). Risk malignancy index was calculated as 159. Following with multidetector contrast-enhanced CT scan showed evidence of cystic lesion of 34.1 cm x 29.9 cm x 22.8 cm with multiple septations and solid component in in postero-lateral aspect, bilateral ovaries could not be differentiated from the mass, suggestive of ovarian cystadenoma with high likelihood of malignancy (Figure 4). In order to relieve the patient of the symptoms, laparotomy was planned. Preoperatively, patient was transfused with two PRCs and started on tablet amlodipine 5 mg twice daily.

**Figure 5:**
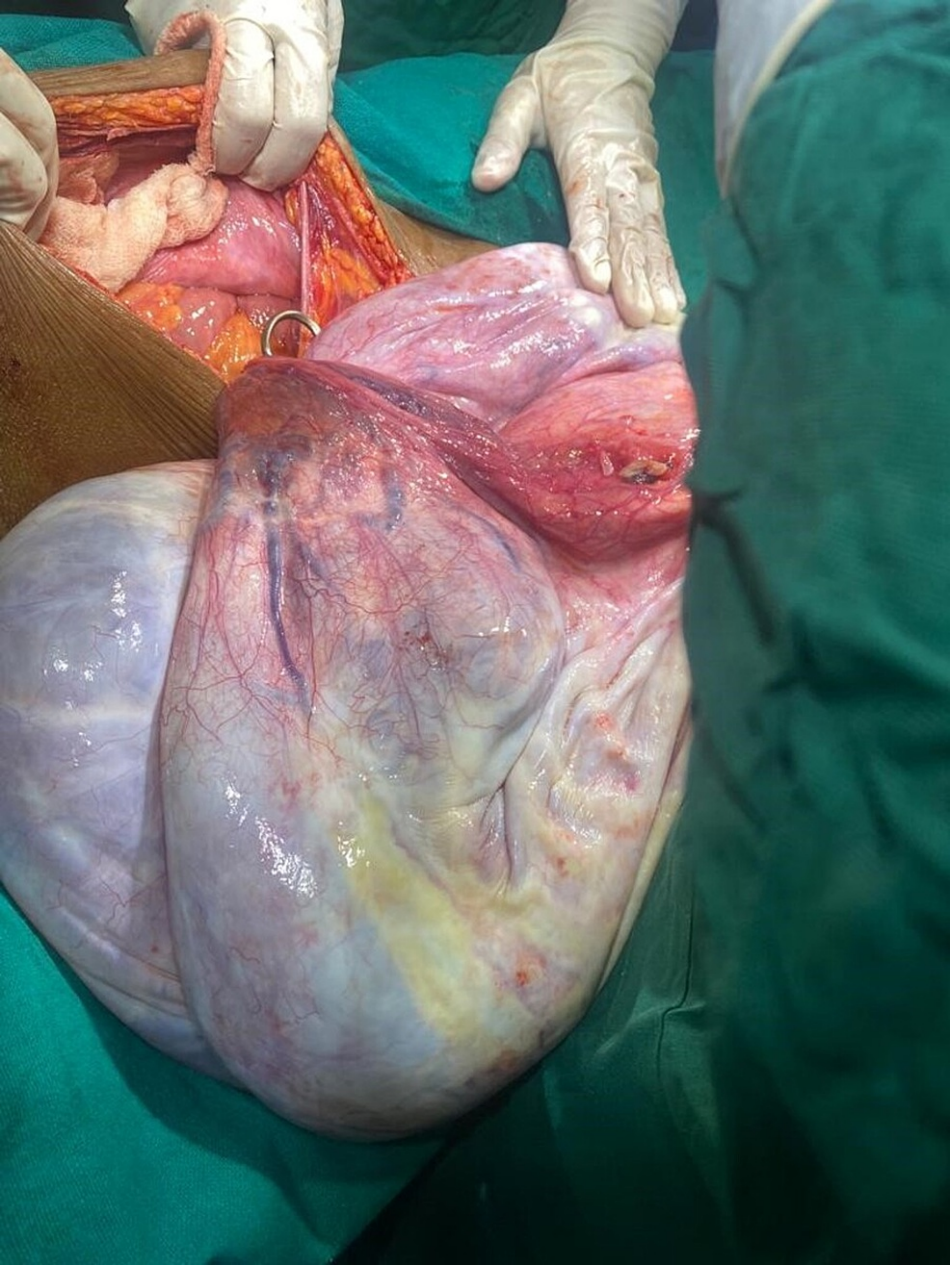
Preoperative dimension of the tumor after aspiration of fluid

**Figure 6:**
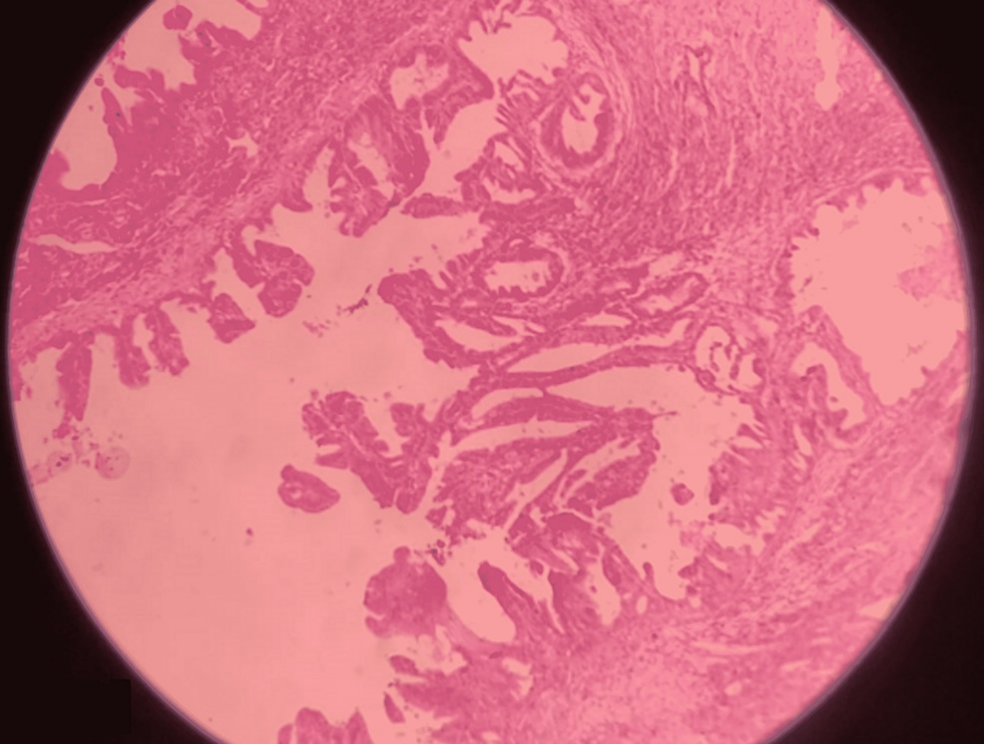
Slide suggestive of borderline mucinous tumor of ovary, on histopathology

After taking informed very high-risk consent with counselling about intraoperative post operative complications such as sudden hypotension and ventilatory support amongst others, staging laparotomy was performed under general anesthesia, with left lateral tilt to the operating table. Abdomen was opened in layers with infraumbilical midline incision. Minimal ascites was present in peritoneal cavity, grossly whitish glistening capsule of tumor was identified which was extending up to xiphisternum, no adhesions of the mass was present in the peritoneal cavity, size of mass was identified as 36 x 30 x 18 cm, mass could not be removed en bloc due to large size of it (Figure 5). Following which fluid from four different quadrants aspirated slowly and sequentially, pyogenic fluid was aspirated from the left quadrant while straw colored fluid was aspirated from other quadrants, a total of 18.5 liters of fluid was aspirated, care was taken to avoid intraperitoneal spillage, cytology report of which did not show presence of any malignant cells. Sudden hypovolemia was avoided by concurrent fluid resuscitation with whole blood and voluven and intravenous fluids. Following aspiration of most of the fluid the origin of tumor was seen to be arising from the right ovary, right-sided cystectomy was performed along with hysterectomy and bilateral salpingo-oopherectomy, entire length of bowel was run starting from the ileo-cecal valve, appendix not seen as appendicectomy was performed 10 years back, undersurface of liver and diaphragm examined, specimens from the vaginal cuff, peritoneum and omentum were also sent for histopathology as part of staging laparotomy. Post-operatively patient was managed in the intensive care unit for two days postoperatively, Foley’s catheter was removed on day 3. Specimen on histopathological examination was revealed as borderline mucinous cystadenoma, and the solid component after aspiration of fluid weighed 14 kilograms with multilocular more than unilocular features with low-malignant potential. The patient was discharged on day 10 after an uneventful post-operative period. She weighed 42 kilograms prior to discharge. Follow-up was planned after one month. On histopathological examination, it was reported as borderline mucinous cystadenoma stage 1C weighing 32.5 kilograms in total (Figure 6).

## Discussion

Ovarian cysts more than 10 cm in dimension are labeled as giant ovarian cysts [6]. Mucinous cystadenomas have the potential to grow into huge mass and rarely remain undiagnosed till they become giant ovarian cysts, they are incidentally found on routine physical examination and sonograms. Mucinous tumors constitute of benign (75%), borderline[10%] and malignant lesions[15%] [2]. Giant ovarian tumors generally pose risk due to their location and pressure effects on surrounding structures. They can undergo a malignant transformation as well. Even though their symptomatology is vague they can cause serious complications such as torsion, rupture, and ascites and due to their expanding size lead to higher grades of dyspnea [7]. As in this case as the patient belonged tribal region and lack of medical facilities this tumor was largely misdiagnosed as ascites and treatment was delayed.

Mucinous tumors have a tendency to occur in the fifth or sixth decade of life, although their actual incidence in postmenopausal women remains underreported due to early detection. This patient also first started exhibiting symptoms in her late sixties. It is imperative to calculate to risk malignancy score (RMI) based on the ultrasonographic assessment and levels of CA 125 prior to operating, so as to formulate an optimal surgical plan and further chemo-radiation needs. This patient had an RMI of 159 well below the cut-off level of 200 [5].

Tumor markers have remained the cornerstone while investigating the patient for ovarian tumors, CA 125 and CA 19.9 are quite sensitive to epithelial tumors. Cancer antigen 19‐9 (CA19‐9) raise is predominantly seen in the gastrointestinal, biliary tract, or ovarian malignancies, notably in mucinous form. In this case as well level of 19.9 was well above the normal of 37 U/mL [8].

Management of the patient depends on various factors such as the patient’s age, menopausal status, whether desirous of fertility, nutritional status of the patient, access to medical facilities, and surgeon’s expertise. Patients with ovarian tumors require surgery at some point of time during the course of the disease, that being said careful planning with a multidisciplinary approach toward management both pre and postoperatively by a team of gynecologists, onco-surgeon, anesthetists, intensivists, and dietician leads to positive outcomes. The patient here had a unilateral tumor for which a staging laparotomy was performed, some surgeons advocate removing the tumor en bloc to avoid any peritoneal spillage of fluid which can further lead to pseudomyxedema peritonei, and/or upstage the disease [9], but at times this is not possible due to sheer size and mass of the tumor as in our case where controlled aspiration of fluid was done from various quadrants to reduce the tumor dimension and ease of removal. It also helps in preventing sudden supine hypotension syndrome [10]. Intraoperative drainage of fluid eliminates the occurrence of splanchnic shock which happens when the constrained splanchnic vascular bed proximal to inferior vena cava is relieved with removal of tumor [11]. Supine posture should always be avoided as resulting vena caval compression might lower the cardiac output causing abrupt loss of pulse and cardiac arrest. The successful outcome of this case can be attributed to the meticulous approach of the multidisciplinary team, optimal pre-operative bowel preparation, adequate transfusions of blood and improving nutritional status prior to surgery.

Various cases of giant tumors have reported in the past, the largest on record being 148.6 kg tumor by Spohn [12] while the largest one reported in India is at 56.95 kg [4], the case of tumor present here seems to be the largest documented ovarian tumor in central India to the best of our knowledge, still the occurrence of such tumor quite rare, and hence, their management strategies require meticulous planning and expertise.

## Conclusions

In spite of advances in screening methods available for ovarian masses, rural women still suffer the agony due to lack of facilities and misdiagnosis as seen in this case where the patient was initially misdiagnosed as having ascites. We wish to highlight this case to bring forth the plight of rural women who are deprived of standard health care.
